# Supplementary material for: Magnitude and factors associated with iron supplementation among pregnant women in anemia hot spot regions of Ethiopia: Multilevel analysis based on Bayesian approach
Source: PLoS One. 2024 Nov 1;19(11):e0313116. doi: 10.1371/journal.pone.0313116 (PMC11530071; doi:10.1371/journal.pone.0313116)
Supplement: Attachment — Submitted filename: Reviewers comments for author (3).docx [file pone.0313116.s001.docx]

**Reviewer’s comments and concerns**

I have reviewed the manuscript titled "Magnitude and Factors Associated with Iron Supplementation among Pregnant Women in Anemia Hotspot Regions of Ethiopia." Below are my comments for the authors to consider for revision:

**Abstract**

1. The author should mention the level of statistical significance in the methods section of the abstract.

2. The data analysis model should also be mentioned.

3. The result of the abstract states that the overall magnitude of iron supplementation among pregnant women is 55.5%, but it requires a confidence interval.

4. The conclusion of the abstract overlaps with the result. The author should rewrite it to avoid repetition.

5. When stating that iron supplementation among pregnant women in anemia hotspot regions of Ethiopia is low, the author should clarify how "low" is determined. It would be helpful to compare this magnitude with national or global levels for context.

**Methodology**

6. It is unclear, 2116 was obtained out of how much overall data?

7. The method of data extraction is not clear.

8. The process of data entry and analysis is not clearly explained. It would be helpful to specify the software used for data entry and analysis.

9. The methodology should explain how confounders were controlled.

**Result**

10. The author needs to describe the findings using the figure (number) with the percentage. For example, an overall magnitude of 55.5% (?/2116). Describing the finding only in percent in the main result section is not acceptable.

11. A confidence interval for the overall magnitude of 55.5% is required.

12. In the table, the overall frequency of residence is (867+308=1175), age group (521+520+135=1176), marital status (1087+14+24+10+42=1,177), and sex of household head (886+289=175). It's important to investigate the reasons for the overall iron supplement variations among variables. This indicates a false report, so the author should recalculate it.

13. Table 3 is confusing, and unnecessary analysis was presented. It's unclear why the author included the results of Estimates, SE, Tail_ESS, and Bulk_ESS in the table, as they were not used in the discussion and conclusion. All those columns should be removed. I recommend that the author revise the entire Table 3 and present it as follows:

| **Variables** | **Category** | **Iron supplement** | | **COR (95% Cl)** | **AOR (95% Cl)** | **P- value** |
| --- | --- | --- | --- | --- | --- | --- |
|  |  | **Yes N (%)** | **No N (%)** |  |  |  |
| Residence | Urban | ?(?) | ?(?) | 1 | 1 |  |
|  | Rural | ?(?) | ?(?) | 0.16[0.04-0.63] | 0.16[0.04-0.63] | 0.002 |

**Discussion**

14. There is no discussion about the magnitude of the iron supplement, which is the main objective. I strongly recommend that the author should discuss the findings of the magnitude.

15. In the discussion section, the author only described the study result but did not discuss or compare it with previous study findings. It should be revised.

16. In paragraph 2 of the discussion, it is stated "This finding is supported by studies (25-27)." This is an incomplete sentence. It should be mentioned where these studies were conducted and what they reported.

17. In paragraph 3, it is stated “This finding is supported by studies conducted in India and Tanzania (25, 28).” However, reference 25 does not indicate a study conducted in India, and 28 does not indicate a study in Tanzania. I strongly recommend that the author should cite the references in the correct place; otherwise, this paper is unacceptable.

**Conclusion and Recommendation**

18. Since there are many limitations of the study, the author should recommend future research to address these gaps.
